# Ultrafast demagnetization by hot electrons: Diffusion or super-diffusion?

**DOI:** 10.1063/1.4964892

**Published:** 2016-10-12

**Authors:** G. Salvatella, R. Gort, K. Bühlmann, S. Däster, A. Vaterlaus, Y. Acremann

**Affiliations:** Laboratory for Solid State Physics, ETH Zurich, 8093 Zurich, Switzerland

## Abstract

Ultrafast demagnetization of ferromagnetic metals can be achieved by a heat pulse propagating in the electron gas of a non-magnetic metal layer, which absorbs a pump laser pulse. Demagnetization by electronic heating is investigated on samples with different thicknesses of the absorber layer on nickel. This allows us to separate the contribution of thermalized hot electrons compared to non-thermal electrons. An analytical model describes the demagnetization amplitude as a function of the absorber thickness. The observed change of demagnetization time can be reproduced by diffusive heat transport through the absorber layer.

## I. INTRODUCTION

The ultrafast demagnetization of a ferromagnet (FM)^1–4^ is expected to be caused by both the hot electron gas^5,6^ and the lattice.^7–9^ Due to the high diffusivity of electrons, ultrafast transport effects become relevant.^5,10,11^ This is particularly interesting, as it allows for the generation of intense femtosecond spin current pulses.^12–14^ Ultrafast transport is also of interest as a ferromagnet can be demagnetized by a pulse of hot electrons, as observed by Eschenlohr *et al.*^6^ They demonstrated that a ferromagnet can be demagnetized indirectly by illuminating a (non-magnetic) metallic absorber film (NA) in contact with the ferromagnet (FM). The pump laser light is absorbed in the NA and heats the electron gas. The initial non-thermal electron gas thermalizes and excites the magnetization of the ferromagnet. They have observed that the ferromagnet is still demagnetized without being directly exposed to the pump laser light. Recently, there was a debate, to which extent the ferromagnet is affected by hot electrons or by visible light transmitted through the absorber layer.^15–17^ In addition, it is not clear if the electrons affecting the ferromagnet are diffusive or show super-diffusive or ballistic properties.

In this paper, we investigate hot-electron induced demagnetization by measuring the demagnetization as well as the non-magnetic polarization contrast as a function of the absorber layer thickness. This way, we can distinguish the effect of electron-mediated, lattice-mediated, and optically induced heating on the demagnetization.

## II. EXPERIMENTAL SETUP

**FIG. 1.**
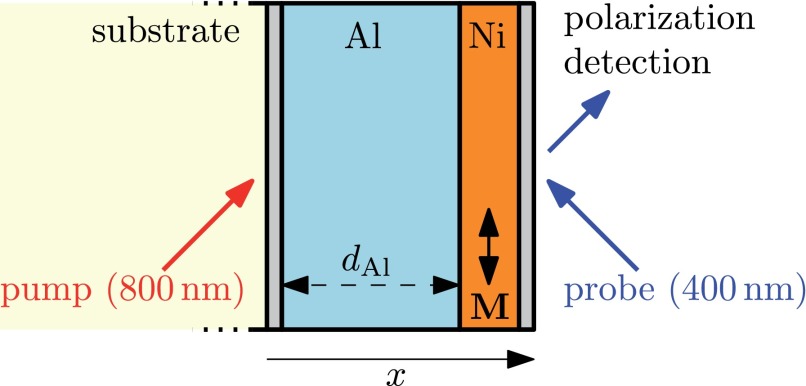
Experimental setup: The sample is excited from the backside by the pump beam through the fused silica substrate. The pump pulse illuminates an Al layer of variable thickness $d_{\text{Al}}$. The ferromagnet on the front side of the sample is probed by the magneto-optical Kerr effect in longitudinal geometry.

We employ the magneto optical Kerr effect (MOKE) in longitudinal geometry to access the optically induced magneto-dynamics of a 10 nm nickel film grown on top of an aluminum film of thickness $d_{\text{Al}}$, see Figure 1. The sample is grown by e-beam evaporation on a glass substrate. The layer structure is 3 nm Ti/$d_{\text{Al}}$ nm Al/10 nm Ni/3 nm Ti. $d_{\text{Al}}$ is varied between 0 nm and 60 nm. The experiment is performed in a pump-probe setup.^1^ An amplified femtosecond laser system provides 800 nm pulses with a repetition rate of 10 kHz. The pulses' length is approx. 25 fs FWHM.

The MOKE is measured by frequency doubled probe pulses (400 nm) to circumvent state blocking effects.^18,19^ In order to observe ultrafast transport, the pump pulses excite the sample from the backside through the glass substrate, whereas the probe pulses detect the magnetization on the front side of the sample. The angle of incidence is $45^{{}^{\circ}}$ for the pump- and probe beam. During the experiment, a magnetic field H=±100 Oe is applied. This field is sufficient to fully saturate the magnetic film in either the up or down direction (↑,↓) in the plane of the sample. Lock-in detection is used to measure the pump-induced change of the polarization rotation $\Delta \theta_{↑,↓}(t)$ by a balanced photodiode detector. Therefore, the pump beam is mechanically chopped at a frequency of 83 Hz. In addition, a λ/4-plate is used before the balanced detector to suppress spurious elliptic contributions. The pump-induced change of the magnetization is determined as

$$\Delta M(t)\propto \Delta \theta_{↑}(t)-\Delta \theta_{↓}(t).$$ (1)

At the same time, we can detect the non-magnetic contribution of the pump-pulse-induced polarization rotation as

$$\Delta N(t)=\Delta \theta_{↑}(t)+\Delta \theta_{↓}(t).$$ (2)

## III. RESULTS

**FIG. 2.**
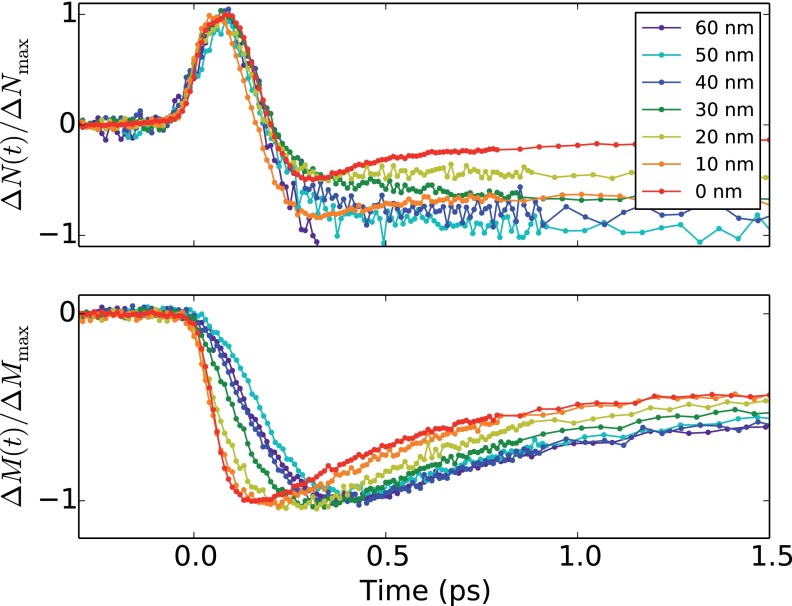
(a) Non-magnetic contrast ΔN(t) for different absorber film thicknesses, scaled to the maximum amplitude of each trace. (b) Demagnetization ΔM(t) scaled to the maximum amplitude of each trace. The temporal overlap has been determined for each sample by the time where $\Delta N(t)/\Delta N_{\text{max}}=0.5$.

**FIG. 3.**
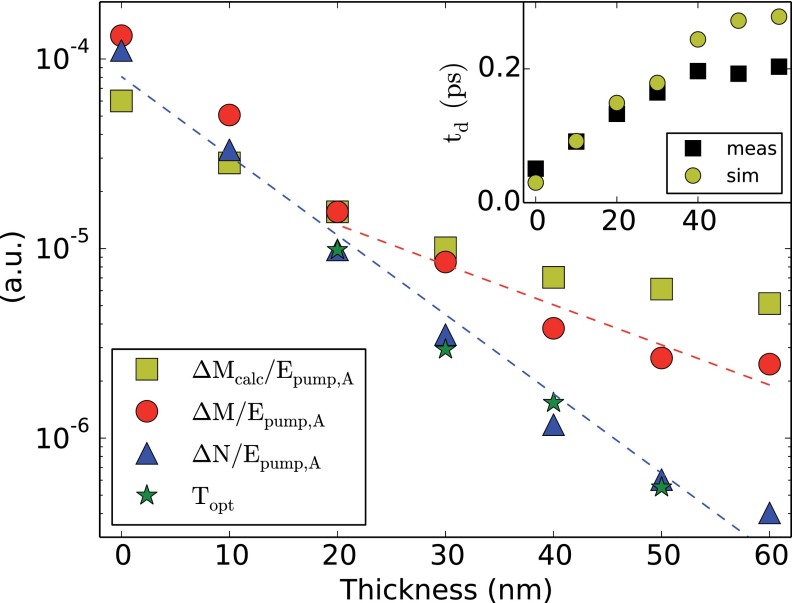
Amplitude dependence of the ultrafast demagnetization (red) and the non-magnetic contrast (blue) as a function of the absorber film thickness $d_{\text{Al}}$, scaled by the pump pulse energy. The non-magnetic contribution follows the optical transmission. The demagnetization initially follows the non-magnetic signal but decays on a longer length scale of 23.5 nm for $d_{\text{Al}}>30\;\text{nm}$. The inset shows the demagnetization time as a function of $d_{\text{Al}}$ for the measurement and the simulation.

In Figure 2, we show the relative demagnetization $\Delta M(t)/\Delta M_{\text{max}}$ as well as the relative non-magnetic polarization change $\Delta N(t)/\Delta N_{\text{max}}$ within the first 1.5 ps for different absorber film thicknesses. In order to be able to compare details of the magnetic response, the pump pulse energy was adjusted such that the ultrafast demagnetization amplitude is within the linear response regime (<10%)^20^ but reaches at least 4% of the saturation magnetization. The linearity has been verified on the samples used in this experiment. The pump pulse energy per area as well as the resulting demagnetizations is shown in Table I. The shape of the ultrafast part of $\Delta N(t)/\Delta N_{\text{max}},\;(t<200\;\text{fs})$ is independent of $d_{\text{Al}}$. On the other hand, the demagnetization $\Delta M(t)/\Delta M_{\text{max}}$ is faster for thinner Al film thicknesses. In order to determine the demagnetization time, *M*(*t*) was fitted by a double exponential function. Then, the demagnetization time from 10% to 90% of the $\Delta M_{\text{max}}$ is determined from the fit. There is a significant increase of the demagnetization time *t_d_* with increasing aluminum thickness, as shown in the inset of Figure 3.

**TABLE I.** Pump pulse energy per area ($E_{\text{pump},A}$) and the resulting demagnetization $\Delta M/M_{s}$.

$d_{\text{Al}}$ (nm)	$E_{\text{pump},A}$ ($\text{mJ}/{\text{cm}}^{2}$)	$\Delta M/M_{s}$ (%)
0	0.21	8.8
10	0.35	5.5
20	1.55	5.2
30	2.97	5.5
40	6.43	5.3
50	8.59	4.9
60	10.77	4.1

As the experiment was performed on individual samples, it was necessary to determine the temporal overlap between the pump and probe pulses experimentally. For temporal alignment, we use the point, where $\Delta N(t)/\Delta N_{\text{max}}=0.5$, as the shapes of the rising edges are independent of $d_{\text{Al}}$. The demagnetization times strongly depend on the sample thickness; however, we cannot see a significant delay in the onset of ΔM as described by Vodungbo *et al.*^17^

In order to identify the causes of ΔM(t) and ΔN(t), we investigate their amplitudes as a function of $d_{\text{Al}}$. In Figure 3, $\Delta N_{\text{max}}/E_{\text{pump},A}$ and $\Delta M_{\text{max}}/E_{\text{pump},A}$ are plotted as a function of the Al layer thickness $d_{\text{Al}}$ ($E_{\text{pump},A}$ is the pump pulse energy per unit area). In comparison, the optical transmission through the film is shown (at an incident angle of $45^{{}^{\circ}}$). Notice that $\Delta N_{\text{max}},\;\Delta M_{\text{max}}$, and the absorption of the sample are in arbitrary units. They have been scaled in amplitude for comparison. The optical transmission matches the decay of $\Delta N_{\text{max}}(d_{\text{Al}})$ on a length scale of 10.5 nm for all measured sample thicknesses. In contrast, the demagnetization $\Delta M_{\text{max}}(d_{\text{Al}})$ first follows the non-magnetic contrast for $d_{\text{Al}}<30$ nm. At larger thicknesses, it decays on a longer length scale of 23.5 nm.

## IV. SIMULATION OF THE DEMAGNETIZATION TIMES

The demagnetization time has been simulated by solving the heat diffusion equation within the electron gas, coupled to the lattice^21^

$$\partial_{t}(\gamma T_{e}^{2})=k(x)\Delta T_{e}-G(x)(T_{e}-T_{l})+P(t,x),$$ (3)

$$c_{l}(x)\partial_{t}T_{l}=G(x)(T_{e}-T_{l}).$$ (4)

Here, *T_e_* and *T_l_* are the electron and lattice temperatures, *P*(*t*, *x*) is the absorbed laser power density, *G* the lattice-electron coupling constant, $\gamma T_{e}$ the heat capacity of the electron gas, *c_l_* the heat capacity of the lattice, and *k* the electron gas' thermal conductivity. The values used in this paper are $\gamma_{\text{Al}}=91.2\times 10^{-27}\;J/{\text{nm}}^{3}{\;K}^{2},\;\gamma_{\text{Ni}}=1077.4\times 10^{-27}\;J/{\text{nm}}^{3}{\;K}^{2},\;G_{\text{Al}}=2.4\times 10^{-22}\;J/{\text{ps}\;\text{nm}}^{3}\;K$ and $G_{\text{Ni}}=10^{-21}\;J/{\text{ps}\;\text{nm}}^{3}/K,\;k_{\text{Al}}=2.3\times 10^{-19}\;J/\text{ps}\;\text{nm}\;K$, and $k_{\text{Ni}}=0.8\times 10^{-19}\;J/\text{ps}\;\text{nm}\;K$ for room temperature.^22,23^ As we are only interested in the initial temperature rise, we neglect cooling of the lattice as well as the lateral heat diffusion. The thickness $d_{\text{Al}}$ affects the simulation in various ways. The absorbed pulse energy profile *P*(*x*) depends on it, as well as the *x* dependence of *k*, *G*, and *c_l_*. The electron gas is heated by two effects: the laser light directly heats the electron gas through absorption, leading to *P*(*t*, *x*) in Equation (3). We assume this effect to immediately affect the electron gas temperature as the creation of electron-hole pairs happens on a sub 10 fs time scale. As $d_{\text{Al}}$ is increased, the contribution of the directly absorbed laser light within the ferromagnet is reduced due to absorption in the Al layer. However, more heat is generated within the Al layer, which eventually diffuses into the Ni layer. This heat transport process is slower as the Al thickness increases.

From the solution of the heat diffusion equation, we estimate the resulting demagnetization to be proportional to the rise of the heat of the electron gas. This is justified, as, for small demagnetization amplitudes, the demagnetization is proportional to the pump pulse energy.^20^ In addition, this proportionality was experimentally verified on the Ni film used in this experiment. This finding is in line with our calculations based on the model from Koopmans^9^ for type I materials and demagnetization amplitudes up to 10%. In 3d ferromagnets, the demagnetization initially follows the heat within the electron gas, as the electron-spin coupling is strong.^9^ The heat per unit volume *V* stored in the electron gas is $\gamma T_{e}^{2}$. The change of the heat within the electron gas caused by a temperature rise from *T*_0_ to *T_e_* is $\Delta Q/V=\gamma (T_{e}^{2}-T_{0}^{2})$. Therefore

$$\Delta M\propto (T_{e}^{2}-T_{0}^{2}).$$ (5)

We used the simulated temperature in the middle of the Ni film. From the simulated rising edge of the demagnetization, we determine the demagnetization time *t_d_* as the 10%–90% rise time. The result is visible in the inset of Figure 3.

## V. ANALYTICAL MODEL OF THE DEMAGNETIZATION AMPLITUDE

The thickness dependence of the demagnetization amplitude can be estimated by a simple analytical model. Here, we do not intend to study the temporal characteristics of the temperature rise of the electron gas. Instead, we develop a simple model, which estimates the electron gas temperature *after* the experimentally determined demagnetization time *t_d_*. The goal of this calculation is to demonstrate that laser light absorption, temperature equilibration within the electron gas, as well as heat conduction from the electron gas to the lattice can account for the observed thickness dependence of the demagnetization amplitude. The heat deposited by the laser pulse per unit area $Q_{\text{dep},A}$ is given by

$$Q_{\text{dep},A}=E_{\text{pump},A}a.$$ (6)

Here, $E_{\text{pump},A}$ is the pump pulse energy per unit area and $a(d_{\text{Al}})$ the absorption of the film. $a(d_{\text{Al}})=E_{\text{absorbed},A}/E_{\text{pump},A}$ has been determined by taking into account the multiple reflections between the film surfaces.^24^ We use the refractive indexes for bulk Al and Ni.^25^ Immediately after the pump pulse (assumed to be infinitely short, ending at *t* = 0), the heat stored in the electron gas is

$$Q_{\text{dep},A}=\int_{0}^{\tilde{d}}\gamma (x)(T_{e}^{2}(x,t=0)-T_{0}^{2})\;dx$$ (7)

with $\tilde{d}=d_{\text{Al}}+d_{\text{Ni}}$ and $T_{0}=300\;K$ the temperature before the pulse. We assume that heat diffusion thermalizes the electron gas within the observed demagnetization time *t_d_*. During this time, the heat is partially transferred from the electron gas to the lattice. With the electron-phonon coupling constant *G*, we get

$$\partial_{t}Q_{\text{el},A}=-\int_{0}^{\tilde{d}}G(x)(T_{e}(x,t)-T_{0})dx.$$ (8)

Here, we assume that the lattice heat capacity is much larger than the heat capacity of the electron gas. Therefore, the lattice temperature does not change. *G* is a function of the position *x*, as *G* is material-dependent. Assuming thermalization, we obtain

$$\partial_{t}Q_{\text{el},A}\approx -\bar{G}\tilde{d}({\bar{T}}_{e}(t)-T_{0})$$ (9)

with $\bar{G}=(G_{\text{Al}}d_{\text{Al}}+G_{\text{Ni}}d_{\text{Ni}})/\tilde{d}$.

The total heat of the electron gas (Eq. (7)) can be further simplified, assuming equilibration along *x* and with the ansatz $T_{e}(t)-T_{0}\propto e^{-t/\tau }$,

$$\partial_{t}Q_{\text{el},A}\approx -\frac{2T_{0}}{\tau }\bar{\gamma }\tilde{d}(T_{e}(t)-T_{0})$$ (10)

with $\bar{\gamma }=(\gamma_{\text{Al}}d_{\text{Al}}+\gamma_{\text{Ni}}d_{\text{Ni}})/\tilde{d}$. Notice, that this is only valid for small $T_{e}(t)-T_{0}$. Combining Eqs. (9) and (10), this leads to

$$\tau =2T_{0}\frac{\gamma_{\text{Al}}d_{\text{Al}}+\gamma_{\text{Ni}}d_{\text{Ni}}}{G_{\text{Al}}d_{\text{Al}}+G_{\text{Ni}}d_{\text{Ni}}}.$$ (11)

With *τ*, the known demagnetization time *t_d_* and Equation (5), we can estimate the demagnetization amplitude in arbitrary units using

$$T_{e}\approx \left(\sqrt{\frac{Q_{\text{dep},A}}{\bar{\gamma }\tilde{d}}+T_{0}^{2}}-T_{0}\right)e^{-t_{d}/\tau }+T_{0},$$ (12)

$$\Delta M\propto (T_{e}^{2}-T_{0}^{2}).$$ (13)

The time *t_d_* is taken from the measured demagnetization time, see Figure 3.

## VI. DISCUSSION

The time dependence of the non-magnetic contribution ΔN(t) is independent of the absorber thickness (for t<250 fs). If we look at its amplitude as a function of $d_{\text{Al}}$, it decays on the same length scale as the optical transmission through the film. It has been demonstrated^26,27^ that linearly polarized light can temporarily cause birefringence in a metal, causing an all-optical (non-magnetic) Kerr rotation for the probe beam. This so-called Specular Optical Kerr Effect (SOKE) is caused by hot electrons, which are far from equilibrium. These carriers are short-lived.^27^ The time scale of the first peak of ΔN corresponds to the results presented by Kruglyak *et al.*^27^ As the amplitude of ΔN decays with $d_{\text{Al}}$ the same way as the optical transmission (see Figure 3), we conclude that ΔN is mainly caused by electrons, which are excited *directly* by the pump beam (and have not been transported from the absorber layer to the Ni layer). Therefore, the SOKE signal is a good reference for the temporal overlap between pump and probe pulses *t*_0_.

For $d_{\text{Al}}<30\;\text{nm}$, the ultrafast demagnetization amplitude follows $\Delta N_{\text{max}}(d_{\text{Al}})$. For thicker films, the decay of $\Delta M_{\text{max}}(d_{\text{Al}})$ happens on a longer length scale of 23.5 nm. We interpret this behavior by considering transport of heat from the Al layer to the Ni layer by the electron gas. The ultrafast demagnetization time depends on the Al thickness, in contrast to the rise time of ΔN. This indicates that heat transport by the electron gas is relevant for the demagnetization. The increase of the demagnetization time caused by diffusion of the electrons is reasonably described by solving the diffusion equation, except for larger film thicknesses. The deviation may be caused by defects in the Al absorber film, which may provide a direct pathway for the pump beam to the Ni layer.

The demagnetization amplitude is qualitatively reproduced by the simple model based on total thermalization as well as transport of heat to the lattice (which is described as a reservoir with constant temperature). The deviation for $d_{\text{Al}}<20\;\text{nm}$ is likely caused by the approximation in Equation (9): for short demagnetization times, the exact shape of the temperature distribution needs to be taken into account.

## VII. CONCLUSIONS

A ferromagnet can be demagnetized indirectly by optical pumping through a normal metal. Two regimes can be identified: For an Al absorber thickness $d_{\text{Al}}\leq 30\;\text{nm}$, the optical transmission through the Al film dominates over heat transport through the electron gas. In contrast, for $d_{\text{Al}}>30\;\text{nm}$, the Ni film is demagnetized by heat transport through the electron gas. The non-magnetic, optically-induced Kerr effect (SOKE) can be observed as well, and decays on the same length scale as the optical absorption. The diffusive transport causes a longer demagnetization time *t_d_*. The thickness dependence of *t_d_* can be reproduced by solving the diffusion equation. The demagnetization amplitude can be calculated by a simple analytical model, assuming rapid thermalization within the electron gas and heat conduction to the lattice. Superdiffusive transport effects have been observed in the past,^11^ but they are not observable in our experiment, as we only see the total effect of heat transport on the magnetization. Diffusion is, at the thickness range investigated in this paper, a good approximation to describe the indirect demagnetization through an absorber layer.
