# Erratum: “Ultrafast demagnetization by hot electrons: Diffusion or super-diffusion?” [Struct. Dyn. **3**, 055101 (2016)]

**DOI:** 10.1063/1.4975037

**Published:** 2017-01-25

**Authors:** G. Salvatella, R. Gort, K. Bühlmann, S. Däster, A. Vaterlaus, Y. Acremann

**Affiliations:** Laboratory for Solid State Physics, ETH Zurich, 8093 Zurich, Switzerland

The total heat of the electron gas per volume was taken as $\gamma (T_{e}^{2}-T_{0}^{2})$, whereas the correct value is $\frac{1}{2}\gamma (T_{e}^{2}-T_{0}^{2})$. The corrected diffusion equation reads

$$\gamma T_{e}\partial_{t}T_{e}=k(x)\Delta T_{e}-G(x)(T_{e}-T_{l})+P(t,x).$$ (3)

The simulation was calculated correctly in the paper.^1^ In Section V (Analytical Model), the following equations need to be adapted accordingly:

$$Q_{\text{dep},A}=\frac{1}{2}\int_{0}^{\tilde{d}}\gamma (x)(T_{e}^{2}(x,t=0)-T_{0}^{2})\;dx,$$ (7)

$$\partial_{t}Q_{\text{el},A}\approx -\frac{T_{0}}{\tau }\bar{\gamma }\tilde{d}(T_{e}(t)-T_{0}),$$ (10)

$$\tau =T_{0}\frac{\gamma_{\text{Al}}d_{\text{Al}}+\gamma_{\text{Ni}}d_{\text{Ni}}}{G_{\text{Al}}d_{\text{Al}}+G_{\text{Ni}}d_{\text{Ni}}},$$ (11)

$$T_{e}\approx \left(\sqrt{\frac{2Q_{\text{dep},A}}{\bar{\gamma }\tilde{d}}+T_{0}^{2}}-T_{0}\right)e^{-t_{d}/\tau }+T_{0}.$$ (12)

These changes affect the result of the analytical model presented in Figure 3. The correct model is in better agreement with the experimental data.

**FIG. 3.**
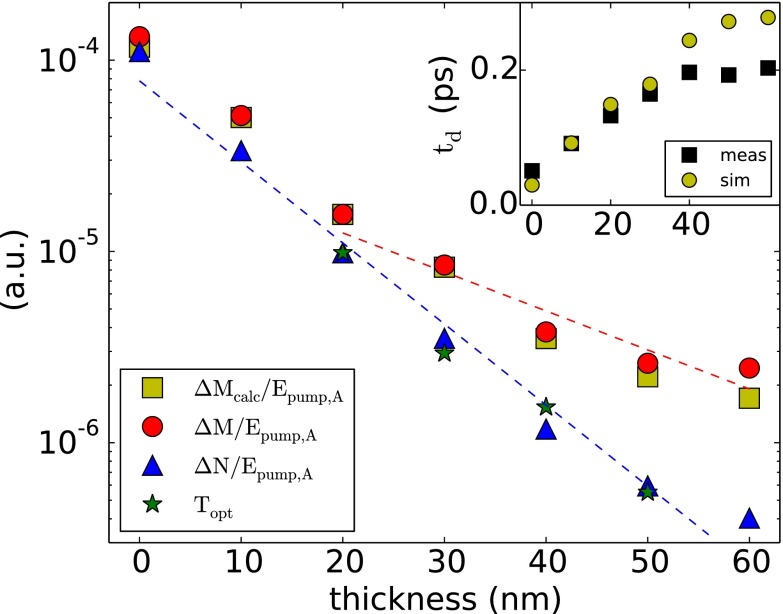
Amplitude dependence of the ultrafast demagnetization (red) and the non-magnetic contrast (blue) as a function of the absorber film thickness *d*_Al_, scaled by the pump pulse energy. The non-magnetic contribution follows the optical transmission. The demagnetization initially follows the non-magnetic signal but decays on a longer length scale of 23.5 nm for *d*_Al_ > 30 nm. The inset shows the demagnetization time as a function of *d*_Al_ for the measurement and the simulation.
